# Phase 3 Clinical Protocol: Placebo‐Controlled, Double‐Blind, Parallel‐Group to Study Safety and Efficacy of NA‐831 in Combination with Lecanemab in Subjects with Early Alzheimer’s Disease

**DOI:** 10.1002/alz.095755

**Published:** 2025-01-09

**Authors:** Lloyd Tran, Zung Vu Tran, Fern Vu

**Affiliations:** ^1^ Biomed Industries, Inc., San Jose, CA USA

## Abstract

**Background:**

Lecanemab has been approved by the FDA for patients with early Alzheimer’s disease (AD). NA‐831 is an experimental drug that has showed a proof of safety and efficacy in Phase 2 clinical trial for patients with early Alzheimer’s disease.

AD patients are treated with a number of comedications not only to address cognitive impairment (standard‐of‐care) but also other comorbidities. It has been shown that amyloid reduction at best leads to modest changes in clinical deterioration and that there is room for additional therapeutics to improve patient quality of life. In addition, amyloid‐related imaging abnormalities side‐effects need to be considered opening the possibilities of dose‐sparing with new combinations.

**Method:**

The phase 3 study consists of a Core and Open Label Extension (OLE) Phase will be conducted to evaluate the efficacy and safety of combination therapy of NA‐ 831 and lecanemab with patients with Early Alzheimer’s Disease (EAD). The Core is an 18‐month treatment, multi‐centers, double blind, placebo controlled parallel group study.

Enrollment: 600 participants. Ages: 50 to 90 Years. All sexes are eligible for study.

Core Study:

Participants will be divided in 3 groups, randomly assigned in a 1:1 ratio to receive a drug or a combination of two drugs or placebo.

Group 1: will receive one 30 mg of NA‐831 capsule orally once a day or placebo,

Group 2: will receive and intravenous lecanemab (10 mg per kilogram of body weight every 2 weeks) or placebo.

Group 3: will receive one 30 mg of NA‐831 capsule orally once a day and intravenous lecanemab (5 mg per kilogram of body weight every 2 weeks) or placebo.

Open Label Extension Phase: Participants completing the core study will receive one 30 milligram (mg) NA‐31 capsule orally once a day, and intravenous lecanemab (5 mg per kilogram of body weight every 2 weeks)

**Result:**

Results will be announced upon the completion of the Phase 3.

**Conclusion:**

The Phase 3 clinical trial will be conducted more than 30 sites in the US and several countries. The details of the Phase 3 methodology and protocol will be presented and discussed.

